# Pitfalls to be considered on the metabolomic analysis of biological samples by HR-MAS

**DOI:** 10.3389/fchem.2014.00033

**Published:** 2014-05-30

**Authors:** Vicent Esteve, Beatriz Martínez-Granados, M. Carmen Martínez-Bisbal

**Affiliations:** ^1^Department of Physical Chemistry, University of ValenciaValencia, Spain; ^2^Biomedical Research Networking Center in Bioengineering, Biomaterials and Nanomedicine (CIBER-BBN)Valencia, Spain

**Keywords:** HR-MAS NMR, metabolomics/metabolite profiling, biological samples, NMR spectroscopy, ERETIC

HR-MAS (High-Resolution Magic Angle Spinning) is considered a powerful technique for metabolomic studies of biological samples that provides “intact” tissue spectra (Cheng et al., 1998; Waters et al., 2000; Sitter et al., 2002; Martínez-Bisbal et al., 2004; Payne et al., 2006; Coen et al., 2007; Bathen et al., 2010). The performance of HR-MAS, followed by quantitative histopathology has demonstrated that, despite some changes, HR-MAS can preserve approximately the tissue histopathologic features producing well-resolved spectra of cellular metabolites (Cheng et al., 2000). Nevertheless, there are some aspects aroused in the literature about the possible biochemical and structural changes that can occur during the sample storage and handling or measurement, and that should be considered in the use of HR-MAS for the metabolical characterization of biological samples. Ignoring these aspects might hamper the rationale use of HR-MAS and a proper interpretation of the results, while considering these factors may contribute to improve the spectral quality and significance and would help to obtain coherent and reliable information of the sample.

In the case of samples of biomedical interest, as those obtained from human patients, when NMR installations are in close proximity to the surgical unit, HR-MAS spectrum of fresh tissue can be obtained within minutes of excision (Kinross et al., 2011). Frequently this is not the case and, in the general practice, the sample needs to be preserved until the moment of the HR-MAS study. The preservation is the key in biological samples, because it is needed to be representative of the alive system. Then, the sample handling and storage conditions are very relevant. The postmortem interval, the time before being preserved and the surgical procedure itself, may promote changes in the tissue metabolite concentration (Cheng et al., 1997; Opstad et al., 2008). This degradation could be observed for example in human brain tissue by the decomposition of NAA to Acetate and Aspartate (Cheng et al., 1997). Therefore, the total sum of NAA and Acetate could represent an estimation of the initial content of NAA, although Acetate could be produced also by other degradation processes (Cheng et al., 1997). In normal rat brain significant changes due to controlled delays in sample freezing were observed in metabolites associated with glycolysis, as Alanine, Glucose, and Lactate (Opstad et al., 2008). Usually, the tissue is preserved by snap-freezing or ultrafreezing until the acquisition of HR-MAS experiments. But freezing and thawing is likely to cause unpredictable amount of cell damage and lysis, with physical disruption of the cellular compartmentalization and changes in the molecular composition (Middleton et al., 1998; Waters et al., 2000; Bourne et al., 2003). Denaturation of proteins, together with the effect of cellular breakdown, will have the combined effect to reduce the number of non-specific binding sites for small endogenous solutes, causing an increment in the concentration of NMR visible metabolites (Middleton et al., 1998). These alterations have been observed also working with other tissues. Thus, the spectra of rat renal tissue after freezing by both, snap-freezing or using liquid nitrogen, compared with spectra of fresh tissue, showed variation in the signal intensities of water soluble metabolites (as Alanine and Glycine) and lipids, and causing a release of specific amino acids as Leucine, Valine and Isoleucine (released from specific binding sites of storage forms), while also reducing the free concentration of renal osmolytes (probably due to enzymatic conversion, binding to macromolecules of sequestration by lipid vesicles) (Middleton et al., 1998; Waters et al., 2000). These changes likely depend on the type of tissue and organ, since following snap-freezing procedures, little changes were observed in rat liver tissue (Waters et al., 2000). Preserving the samples in a buffer and frozen until the NMR study or flushing the samples with D_2_O or saline D_2_O before being introduced in the rotors may result in the loss of large and unpredictable amounts of possibly diagnostic metabolites (around 40–50% of the metabolites would be found in the storage buffer) and would affect to the metabolite absolute quantization (Bourne et al., 2003).

The last step in the sample preparation has been usually the addition of substances as internal patron for chemical shift referencing and absolute quantification. This practice could be useful in the study of liquid extracts, since the external reference can be introduced in a capillary in the NMR tube, isolated from the sample. Nevertheless, in the study of biological samples by HR-MAS these substances are in the same compartment than the sample and some of them have been demonstrated to interact with the sample (Kriat et al., 1992; Payne et al., 2006; Albers et al., 2009; Martinez-Bisbal et al., 2009; Esteve et al., 2012). Owing of this, standard reference compounds like DSS (2,2-dimethyl-2-silapentane-5-sulfonate sodium salt) and TSP [3-(trimethylsilyl)propionic-2,2,3,3-d(4) acid] have shown a reduction in their intensity, causing an overestimation of the metabolite concentration when used as a reference for tissue studies (Albers et al., 2009; Martinez-Bisbal et al., 2009). MDPA (methylene diphosphonic acid) used as a reference for ^31^P experiments, has shown changes in the intensity, the width and in the chemical shift depending on the amount of tumor, the pH and the mass of the sample (Esteve et al., 2012). Moreover these effects, the addition of MDPA caused a drop in the pH (Esteve et al., 2012). In order to avoid the use of these substances, alternative methods should be considered. For example ERETIC™ method (electronic reference to *in vivo* concentrations Akoka et al., 1999), either electronically generated during the acquisition (Tessem et al., 2008; Albers et al., 2009; Martinez-Bisbal et al., 2009; Sitter et al., 2010) or synthetically generated in the postprocessing (Ben Sellem et al., 2011; Esteve et al., 2012), has been verified in the evaluation of absolute concentration of biological samples by HR-MAS. Other approach is the use of PULCON principle (pulse length based concentrations determination) which does not need any special hardware or software (Wider and Dreier, 2006) and that has recently been applied to the metabolical quantification of phosphorilated metabolites of breast tumor tissue samples by ^31^P HR-MAS (Esmaeili et al., 2014).

Regarding to the acquisition of the experiments, there are several factors to be considered. The experiments need to be performed at low temperatures to preserve as much as possible the biochemical profile. Moreover, under HR-MAS conditions the large centrifugal forces can damage the structure of the sample. The time required for 1D ^1^H NMR acquisition experiments is of roughly 30 min, which limits considerably the degradation of the tissue. Very often, a histopathology study is performed on biopsy samples that have undergone 1D ^1^H HR-MAS analysis. Nevertheless, the acquisition of 2D NMR spectra can take several hours. Surprisingly, it appears that the prolonged spinning times on HR-MAS may have more significant effects on the metabolite profile than the tissue ischaemia during biopsy excision and the delays in snap-freezing (Opstad et al., 2008). This mechanical damage could be also the major contributor to the degradation of NAA to Acetate and Aspartate, even more than the period of ischaemia (Opstad et al., 2008). Some biochemical changes may be coherent with the sample degradation but, as observed in rat brain tissue, an increment of 18% in the content of Creatine over a 4 h period would be explained by the effects of spinning in the tissue sample and the release of the NMR-invisible Creatine stores (Opstad et al., 2008). The MAS at 5 kHz in cell suspensions has been demonstrated to induce the formation of a two-phase system, composed of an inner cylinder of essentially cell-free water and an outer hollow cylinder of tightly packed cells (Chen et al., 2003). In tissues, MRM (magnetic resonance microscopy) images of rotors after HR-MAS has shown that the tissue was stuck to the rotor wall, under the effect of high spinning speed (Martinez-Bisbal et al., 2011). Moreover, MRM localized spectroscopy has shown a transference of metabolites from the tissue stuck to the wall to the medium in the inner space of the rotor (Martinez-Bisbal et al., 2011), either in D_2_O or in PBS D_2_O, implying a change in the location of an undetermined quantity of metabolites that previously were inside the cells or in the extracellular space. These high spinning speeds have been applied to devoid of spinning sidebands. Nevertheless, the quality of the spectra has been shown to strongly depend on the sample preparation. The rotor volume and the internal sample shape may affect to the spectral quality, since smaller spherical inner cavities seem to improve the resolution and sensitivity owing the symmetrical distribution of the tissue sample (Waters et al., 2000). The presence and intensity of spinning sidebands has been shown to be highly dependent on factors linked to the sample preparation as the position and shape of the sample and the presence of air bubbles (Renault et al., 2013). The incomplete filling of the detected volume is capable to induce a large number of spinning sidebands (Renault et al., 2013). It seems that restricting the sample chamber to a small volume at the center of the coil with the insert located at the top of the rotor drastically reduces the number and magnitude of the spinning sidebands and thus high quality NMR spectra can be obtained at moderate MAS frequencies of 500 Hz (Renault et al., 2013). Other issue are the long times needed for the acquisition of bidimensional spectra, and in this sense, the reduction of the acquisition time is work in progress with the use of non-uniform sampling methods (Renault et al., 2013) and reduced spinning speeds.

The use of standardized protocols for this kind of analysis would be of help since the metabolic changes introduced by the various steps of the analysis (sample collection, sample preparation, NMR acquisition) would be then more reproducible among different samples. It would be advisable to avoid these effects to approach the sample state to the “intact” as much as possible. Nevertheless, assuming that the sample will undergo changes during the tissue resection, handling, storage, and spectra acquisition, the strategy could be searching for a set of metabolical changes that inform about the real state of the tissue. This would serve as a basis to interpret the biochemical profile observed in the tissues and to establish the metabolical changes due to the pathological condition and not to the experimental process. Histological studies of the samples after HR-MAS or the analysis of molecular indicators of cellular integrity might also contribute to estimate the tissue state.

## Conflict of interest statement

The authors declare that the research was conducted in the absence of any commercial or financial relationships that could be construed as a potential conflict of interest.
